# Comparison of Adopted and Nonadopted Individuals Reveals Gene–Environment Interplay for Education in the UK Biobank

**DOI:** 10.1177/0956797620904450

**Published:** 2020-04-17

**Authors:** Rosa Cheesman, Avina Hunjan, Jonathan R. I. Coleman, Yasmin Ahmadzadeh, Robert Plomin, Tom A. McAdams, Thalia C. Eley, Gerome Breen

**Affiliations:** 1 Social, Genetic & Developmental Psychiatry Centre, Institute of Psychiatry, Psychology & Neuroscience, King’s College London; 2 National Institute for Health Research (NIHR) Biomedical Research Centre for Mental Health, South London and Maudsley National Health Service (NHS) Trust, London, United Kingdom

**Keywords:** educational attainment, polygenic scores, gene-environment interplay, adoption

## Abstract

Polygenic scores now explain approximately 10% of the variation in educational attainment. However, they capture not only genetic propensity but also information about the family environment. This is because of passive gene–environment correlation, whereby the correlation between offspring and parent genotypes results in an association between offspring genotypes and the rearing environment. We measured passive gene–environment correlation using information on 6,311 adoptees in the UK Biobank. Adoptees’ genotypes were less correlated with their rearing environments because they did not share genes with their adoptive parents. We found that polygenic scores were twice as predictive of years of education in nonadopted individuals compared with adoptees (*R*^2^s = .074 vs. .037, *p* = 8.23 × 10^−24^). Individuals in the lowest decile of polygenic scores for education attained significantly more education if they were adopted, possibly because of educationally supportive adoptive environments. Overall, these results suggest that genetic influences on education are mediated via the home environment.

An important process by which genes and environments work together to influence behavior is gene–environment correlation (Plomin, DeFries, Knopik, & Neiderhiser, 2016). Gene–environment correlation refers to the association between the genotype that individuals inherit from their parents and the environment in which they are raised (Plomin, DeFries, & Loehlin, 1977). Three forms of gene–environment correlation are typically distinguished: passive, active, and evocative. An example of passive gene–environment correlation is that more educated parents are likely to provide both beneficial genes and educationally supportive family environments, such as books in the home, for their children. Therefore, shared genes confound associations between putative environmental variables and child attainment. Active and evocative gene–environment correlations reflect how genotypes lead to phenotypes: Individuals select and evoke environments on the basis of their genetically influenced traits.

It is essential to investigate gene–environment interplay in educational attainment for several reasons. First, educational attainment is an important trait for individuals and society, partly because of its significance for health and health inequalities. Second, gene–environment correlation clearly matters for educational attainment. Adoption, twin, and instrumental-variables research suggest that shared genes largely explain associations between parent and child attainment (Holmlund, Lindahl, & Plug, 2011). Third, polygenic scores, which index the genetic liability that each individual carries for a specific trait, are notably powerful for educational attainment and now predict approximately 10% of the variation in years of education (Lee et al., 2018), a finding with potential social implications (Plomin & von Stumm, 2018). However, it has been shown only recently that this prediction includes not only direct genetic effects on an individual’s own education but also indirect genetic effects through relatives, that is, predicting the family environment (Bates et al., 2018; Kong et al., 2018).

Behavior-genetic study designs are needed to disentangle causal processes affecting educational attainment. Adoption studies do this by removing overlapping genetic and environmental influences (passive gene–environment correlation). This is achieved by measuring the resemblance of adopted children to their birth parents and to their adoptive parents. The former gives an estimate of direct genetic influence independent of passively correlated environmental effects. The latter gives an estimate of shared environmental influence, free of correlated genetic effects. Passive gene–environment correlation may be estimated as the extent to which genes contribute more to the covariation between measures of the family environment and offspring traits in nonadoptive than adoptive families (Plomin, Loehlin, & DeFries, 1985). Notably, other forms of gene–environment correlation are still present in adoptees, because heritable proclivities lead them to select and evoke experiences.

More recently, researchers have applied genomic tools to family data to estimate direct and indirect effects on educational attainment (Bates et al., 2018; Domingue, Belsky, Conley, Harris, & Boardman, 2015; Kong et al., 2018; Selzam et al., 2019; Wertz et al., 2018; Young et al., 2018). These designs are conceptually related to adoption designs, because they account for shared genes between parents and offspring. For example, genetic variants that were not passed on by parents can have only indirect effects on offspring traits, through genetically influenced parental behavior (Bates et al., 2018; Kong et al., 2018). When indirect effects were controlled for with a polygenic score for education based on nontransmitted variants, the variance explained by the transmitted score shrank from 5% to 2% (Kong et al., 2018). The nontransmitted score also independently predicted attainment. The family environment is an important contributor to polygenic-score prediction because it is adding to estimates of genetic influence and because parents still influence their offspring after analyses control for shared (transmitted) genes.

The present study drew on an unusually large and relatively unexplored sample of adoptees and harmonized a traditional quantitative genetic approach with modern genomic tools. Our main aim was to use the natural experiment created by adoptive placement to measure the importance of passive gene–environment correlation for educational attainment. When children are adopted by nonrelatives, the indirect genetic path between the rearing environment and their traits is not present because adoptive parents are not genetically related to adopted children. Three hypotheses follow. First, the phenotypic variance should be lower in adoptees compared with nonadopted individuals, because adoptees do not have the additional source of variance of passive gene–environment correlation (Loehlin & De Fries, 1987; Plomin, 1994). This discrepancy could also be due to adoptive families varying less in socioeconomic status or being selected for perceived parenting ability (Natsuaki et al., 2019; Rutter, 2006). Second, if passive gene–environment correlation inflates heritability estimates, then heritability should be lower in adoptees than in nonadopted individuals, because adoptees are reared in environments that are less correlated with their genotypes. Third, for the same reason, the variance explained by polygenic scores will be lower in adoptees and may be closer to the direct genetic effect of an individual’s own DNA.

## Method

### Sample, genotype quality control, and phenotype definition

The UK Biobank is an epidemiological resource including British individuals from the ages of 40 to 70 at recruitment (Allen, Sudlow, Peakman, Collins, & UK Biobank, 2014). UK Biobank participants were asked, “Were you adopted as a child?” In total, 7,407 individuals said “yes,” and 495,209 individuals said “no.” No additional information was collected on factors that are understood to reduce the representativeness of adoptees as a study sample: the age of adoption, whether the adoption was domestic or international, or whether individuals were adopted by biological relatives. Genome-wide genetic data came from the full release of the UK Biobank data and were collected and processed according to the quality-control pipeline (Bycroft et al., 2018). We restricted analyses to individuals with full phenotypic data for education, who also passed genotype quality-control criteria. This left 6,311 adopted and 375,343 nonadopted individuals for analysis.

Genotype quality-control criteria were common genetic variants of minor allele frequency greater than 0.01 that were directly genotyped or imputed with high confidence (information-content metric > 0.4), individuals with a genotype call rate greater than 98% who had concordant phenotypic and genetic gender information, and individuals who were unrelated to others in the data set (less than third-degree relatives). We removed relatives using a greedy algorithm to minimize the exclusion of adoptees. To reduce confounding from population stratification, we limited all analyses to individuals of European ancestry, as defined by four-means clustering on the first two genetic principal components provided by the UK Biobank. We also controlled for 10 ancestry principal components of the European sample in all genomic analyses.

Years of education, a proxy for educational attainment, was defined according to International Standard Classification of Education categories, as in previous genomic studies of the phenotype in UK Biobank and other samples (Lee et al., 2018). The response categories were as follows: none of the above (no qualifications) = 7 years of education; Certificate of Secondary Education (CSE) or equivalent = 10 years; O level/General Certificate of Secondary Education (GCSE) or equivalent = 10 years; A level/AS level or equivalent = 13 years; other professional qualification = 15 years; National Vocational Qualification (NVQ), Higher National Diploma (HNC), or equivalent = 19 years; college or university degree = 20 years.

### Statistical analyses

#### Phenotypic comparisons

First, we formally tested the hypothesis that nonadopted individuals show greater phenotypic variance than adopted individuals because of the presence of an additional source of variance (passive gene–environment correlation). A nonparametric test was used given the nonnormal distribution of the years-of-education variable (Brown & Forsythe, 1974). This test is based on absolute deviations from the median rather than the group mean. We also tested for differences in years of education, age, and sex between the two groups using a Wald test, *z* test, and Wilcoxon test, respectively.

#### Single-nucleotide-polymorphism (SNP) heritability estimation

Second, to test the hypothesis that heritability is lower in adoptees, whose rearing environments are less correlated with their genotypes, we estimated the variance explained by common genetic variants for years of education in adoptees using genomic-relatedness-based restricted maximum likelihood (GREML; Yang, Lee, Goddard, & Visscher, 2011) and compared this with the heritability estimate for nonadopted individuals. This method estimates heritability as the extent to which genetic similarity among unrelated individuals can predict their trait similarity. In GREML, a matrix of genomic similarity for each pair of unrelated individuals across genotyped variants is compared with a matrix of their pairwise phenotypic similarity using a random-effects mixed linear model. This allows the variance of a trait to be decomposed into genetic and residual components using maximum likelihood. We used two genetic-relatedness matrices: one for adopted individuals and a second for a subset of 6,500 nonadopted individuals. This was to enable comparison of two similarly sized samples and to reduce the computational burden that results from scaling GREML to a sample as large as the UK Biobank. For both genomic matrices, we used a relatedness cutoff of 0.025. Subsamples were made using the “sample_n” function in the *dplyr* package in the R programming environment (Version 3.5; R Core Team, 2019). We compared these results with heritability estimates derived from a second method, linkage-disequilibrium score regression (LDSC; Bulik-Sullivan, Loh, et al., 2015). Unlike GREML, LDSC does not require individual-level data, allowing it to be computationally feasible to estimate the heritability of education in the full sample of nonadopted individuals. LDSC also enabled us to estimate genetic correlations (see below).

#### Polygenic scoring

Third, we tested whether the power of polygenic scores was greater for individuals who were reared with their biological relatives than for adoptees. The sample of nonadopted individuals was subdivided into three independent groups for polygenic-score analyses. Our first sample consisted of 318,843 nonadopted individuals for genome-wide-association (GWA) analysis. The purpose of this was to estimate the effect sizes of associations between genome-wide genetic variants and years of education for the creation of individual-level polygenic scores. We derived our base summary statistics file for years of education by meta-analyzing summary statistics from our own GWA analysis in this subsample with independent summary statistics obtained from the Social Science Genomics Consortium (excluding UK Biobank and 23andMe; Lee et al., 2018). The sample size for these external summary statistics was 324,160, leading to a total sample size of 643,003 individuals in our GWA meta-analysis.

Our second independent sample included 50,000 individuals for training our polygenic scores for years of education, that is, identifying the optimal *p*-value threshold for inclusion of SNPs. The standard set of *p* values in PRSice 2 software was tested: .001, .05, .1, .2, .3, .4, .5, 1 (Choi & O’Reilly, 2019).

Our third independent sample contained 6,500 individuals, to match the sample size of adopted individuals, on which to run polygenic-prediction models. In these prediction models, we regressed the years-of-education phenotype in the UK Biobank on polygenic scores for years of education in adoptees and then repeated the analysis in the 6,500 nonadopted individuals. In this third set of analyses, we used a set *p*-value threshold obtained from the training step. This exact sample was the same as the one used to estimate SNP heritability of years of education. Notably, this polygenic-score analysis is better powered than the SNP heritability analysis, because it capitalizes on the power of the large discovery sample (*N* = 643,003).

As a negative control, we tested the polygenic-prediction comparison between adopted and nonadopted individuals for height, which has not shown evidence of passive gene–environment correlation in previous studies (Kong et al., 2018; Selzam et al., 2019). As with the education analysis, we trained the polygenic score on the basis of the largest independent association study (Wood et al., 2014) in the sample of 50,000 individuals; we then tested the prediction at the best *p*-value threshold in our two independent and similarly sized samples of adopted and nonadopted individuals.

#### Supplemental analyses

##### Heritability of adoption status

Substantial heritability of our environmental moderator might have affected the interpretation of our main results. To explore this, we also estimated the heritability of adoption status using LDSC in the full sample (*N* = 381,654). The genetic “influence” on adoption largely arises in the biological-parent generation because heritable traits influence the likelihood of adoption of their child.

##### Polygenic-score-by-adoption interaction analyses

Differences in genetic influences on the same trait across contexts—in this case, adoption—can also be conceptualized as gene–environment interaction, whereby the impact of genes on educational attainment may be contingent on adoption status. We aimed to further explore our main results by testing a formal polygenic-score-by-adoption interaction regression model. The model contained main effects for polygenic score for years of education, adoption, and covariates, plus the interaction term, as well as interaction terms for polygenic score and adoption with each covariate (Keller, 2014). We tested a linear model for additive interaction and a logistic model for multiplicative interaction. To visualize any interaction, we plotted the regression slopes for polygenic prediction of educational attainment for adopted and nonadopted individuals (with both variables standardized to have a mean of 0 and a standard deviation of 1). Additionally, we stratified polygenic scores for adopted and nonadopted individuals overall (*N* = 12,811) into deciles and tested for mean differences in years of education between adopted and nonadopted groups in each decile.

##### Qualitative differences in the genetic influence on education by adoption status

We assessed whether education is driven by the same set of genetic influences in adopted and nonadopted individuals. First, we estimated the genetic correlation between years of education in our samples. For this, we ran GWA analyses of years of education in the full sample of nonadopted individuals (*N* = 375,343) and in the sample of adoptees, and then estimated the genetic correlation between them using LDSC. Second, we tested whether education is genetically linked to different traits between adoptees and nonadopted individuals. To this end, we estimated genetic correlations between years of education and 247 traits, available on LD Hub (Zheng et al., 2017), for both adopted and nonadopted individuals. We compared the magnitudes of genetic correlations between years of education and other variables between the adopted and nonadopted samples with *z* tests.

##### Birth-year-related differences in genetic influence

During the period when UK Biobank participants were growing up (1930s–1970s), access to education increased, and there was great change in the norms and regulations surrounding reproduction, contraception, and adoption. Previous studies have found that genetic influence on years of education increased in this period in the United Kingdom, because environmental differences between people had less influence on whether they stayed in education (Lee et al., 2018). We investigated temporal change in patterns of genetic influence on education in adopted compared with nonadopted individuals by stratifying polygenic-prediction analyses according to year of birth. Specifically, all individuals were split into seven mutually exclusive birth-year groups, each with a range of 5 years, and polygenic-score analyses were conducted separately for each of the year groups.

All analyses (SNP heritability, polygenic scoring, GWA) controlled for the following covariates: sex, age, 10 ancestry principal components, and factors capturing genotyping batch and center. The majority of the analyses were completed in R Version 3.5. GREML was performed in the GCTA software (Yang et al., 2011). GWA meta-analysis was performed in METAL (Willer, Li, & Abecasis, 2010). Polygenic-score analyses were performed in PRSice 2 (Choi & O’Reilly, 2019). To compare polygenic-score results between adopted and nonadopted individuals, we obtained bootstrapped standard errors for the *R*^2^ statistics using the boot package in R, with 1,000 replications. Genome-wide genetic correlations were estimated using LDSC (Bulik-Sullivan, Finucane, et al., 2015) and LD Hub (Zheng et al., 2017). The UK Biobank is a controlled-access public data set available to all bona fide researchers.

## Results

### Sample analyzed

The total sample of individuals with education-phenotype data and quality-controlled genotype data was 381,654. As described in the Method section, individuals were split into four mutually exclusive groups: (a) 6,311 individuals adopted as children, (b) 318,843 nonadopted individuals for GWA analysis, (c) 50,000 nonadopted individuals for training of polygenic scores, and (d) 6,500 nonadopted individuals for genomic analyses for comparison with adoptees. Nonadopted individuals were randomly placed into groups b, c, and d.

### Phenotypic results

Phenotypic differences between adoptees and nonadopted individuals were generally modest in size but because of the large sample size in this study, several were statistically significant (see Table 1). We found that there was significantly greater variance in years of education for nonadopted individuals than for adoptees (26.2 vs. 25.8, respectively; *p* = .002 compared with 6,500 nonadopted individuals in group d; *p* = 3.2 × 10^−5^ compared with all nonadopted individuals). Table 1 provides descriptive statistics for years of education, age, and sex in the two groups. Adoptees in the UK Biobank were significantly younger on average (*p* = .026 compared with group d; *p* = .009 compared with all nonadopted individuals), although point estimates were similar (56.4 vs. 56.7). There were significantly more males in the adopted group (*p* = .033 compared with group d; *p* = .008 compared with all nonadopted individuals), but the magnitude of the difference was small (48% vs. 46% male). Adoptees had significantly fewer years of education (*p* = 3.3 × 10^−11^ compared with group d; *p* < 2.2 × 10^−16^ compared with all nonadopted individuals in the UK Biobank). This is also reflected in the lower percentage of college attendees (20 years of education in Table 1) in the adopted group (28% compared with 33% in the nonadopted group). All comparison results were consistent between the large and small samples of nonadopted individuals.

**Table 1.** Descriptive Statistics for Adopted and Nonadopted Individuals Included in the Study

Variable	Adopted (*n* = 6,311)	Nonadopted (*n* = 375,343)	Nonadopted (Group d; *n* = 6,500)
Age	*M* = 56.4, *SD* = 8.53	*M* = 56.7, *SD* = 8.01	*M* = 56.7, *SD* = 8.06
Sex	48% male (*n* = 3,010)	46% male (*n* = 172,706)	46% male (*n* = 2,978)
Years of education			
7	1,209 (19%)	62,651 (17%)	1,064 (16%)
10	1,780 (28%)	100,210 (27%)	1,709 (26%)
13	749 (12%)	43,448 (12%)	755 (12%)
15	350 (6%)	19,428 (5%)	354 (5%)
19	433 (7%)	24,300 (6%)	428 (7%)
20	1,790 (28%)	125,306 (33%)	2,190 (34%)

Note: For years of education, both *n*s and percentages of the subsample are given. Adoptees were compared with the full sample of nonadopted individuals and with our smaller subsample used for genomic analyses (group d).

### SNP heritability estimates

**Fig. 1.**
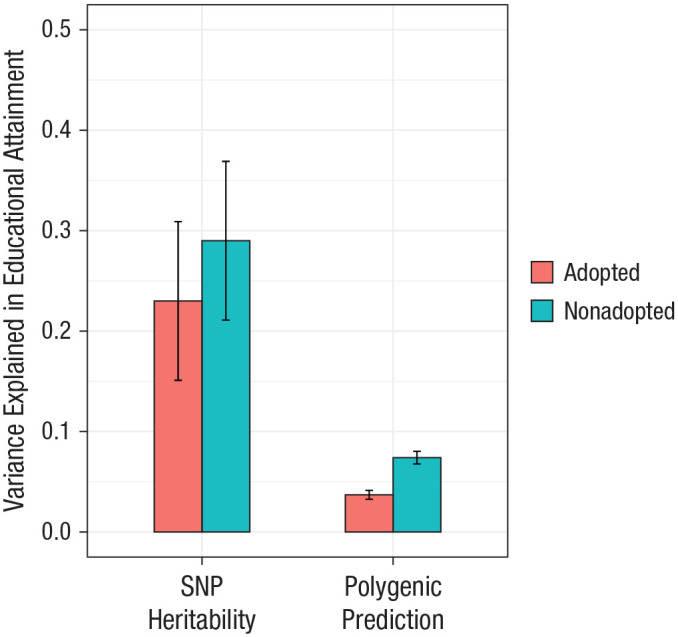
Estimates of the variance explained by common single-nucleotide polymorphisms (SNPs) for years of education and by polygenic scores for education, separately for adoptees and individuals reared with their relatives. Error bars show 95% confidence intervals (CIs). Sample sizes for polygenic-prediction analyses were 6,311 and 6,500 for adopted and nonadopted individuals, respectively; sample sizes for genomic-relatedness-based restricted-maximum-likelihood (GREML) heritability analyses were lower (6,227 for adopted and 6,362 for nonadopted individuals) because relatives were removed at a cutoff of > 0.025. For polygenic-score results, CIs were obtained by bootstrapping with 1,000 replications.

Figure 1 shows GREML-derived SNP heritability estimates for years of education in both adopted individuals and nonadopted individuals (left-hand bars). The estimate of heritability was larger in individuals reared with their relatives (0.29, *SE* = 0.079), compared with adopted individuals (0.23, *SE* = 0.079). However, confidence intervals were wide and overlapped, so the difference in heritability was not significant.

It was not computationally feasible to estimate the heritability of education using all nonadopted individuals with GREML. Notably, though, the LDSC-derived heritability was 0.17 (*SE* = 0.005) in the full sample of nonadopted individuals (*N* = 375,343) and 0.14 (*SE* = 0.073) for adoptees, corroborating the pattern of results found using GREML. LDSC estimates are typically lower than GCTA and GREML-derived estimates (Evans & Keller, 2018).

### Polygenic-prediction results

Figure 1 shows that twice as much phenotypic variance in years of education was explained by polygenic scores for years of education in nonadopted individuals (0.074) as in adoptees (0.037). This difference was highly significant (*p* = 8.23 × 10^−24^). The optimal significance threshold (*p*) for inclusion of SNPs was 1 (see Table S1 in the Supplemental Material available online). Table S2 in the Supplemental Material shows the full results from the polygenic-prediction analyses.

For our negative control analysis of height, as expected, the variance explained by the polygenic score in adoptees (0.127, *SE* = 0.008) versus nonadopted individuals (0.134, *SE* = 0.008) was not significantly different (*p* = .62). The optimal significance threshold (*p*) for inclusion of SNPs in the polygenic score was .001.

### Supplemental analyses

#### Heritability of being adopted

We found a liability-scale SNP heritability of being adopted of 0.059 (*SE* = 0.004), assuming the population prevalence of adoption is identical to the sample prevalence (1.7%). If the actual population prevalence differed and was, for example, 0.7% or 2.7%, the liability-scale SNP heritability would become 0.047 (*SE* = 0.002) or 0.066 (*SE* = 0.005), respectively. Adoption status showed significant genetic correlations with education, age at first birth, depression, and obesity after analyses corrected for multiple testing (see Table S5 in the Supplemental Material), although these correlations should be viewed with caution given the low SNP heritability of adoption. Adoption status could be significantly predicted by the polygenic score for years of education (*R*^2^ = .008, *p* < 2 × 10^−16^). The heritability of adoption was low but may have confounded our between-groups comparisons.

#### Polygenic-score-by-adoption interaction

**Fig 2.**
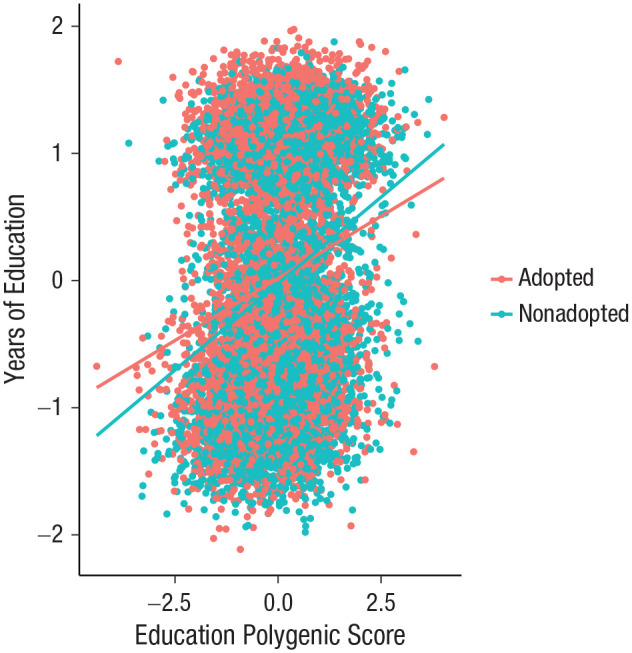
Results of the regression of years of education on polygenic score for education, separately for 6,311 adoptees and 6,500 nonadopted individuals. Best-fitting regression lines are shown for each group, and each data point represents one individual. The two clusters of data points reflect the distinct groups of individuals who did and did not attend university.

We tested a formal interaction model to further examine the finding that genetic influences on education are weaker in the sample of adoptees. The interaction between polygenic score and adoption status in predicting years of education is shown in Figure 2. The regression slope is significantly steeper in the nonadopted group, indicating that years of education increases more as polygenic scores for education increase in this group compared with the adopted group. See Table S3 in the Supplemental Material for results of the full interaction model. Using linear regression, we confirmed that polygenic prediction of education interacts beyond additivity with adoption status (interaction estimate = −0.33; *p* = 2.66 × 10^−4^). This means that polygenic scores had a smaller association with education in adoptees. Then, using logistic regression instead of linear regression, we also found that the interaction exceeded multiplicativity (interaction estimate = −0.18; *p* = .0009). The finding of interaction exceeding both additive and multiplicative models means that the combined effect of polygenic score for education and adoption status is not scale dependent and is greater than either the sum or product of their individual effects, respectively.

**Fig. 3.**
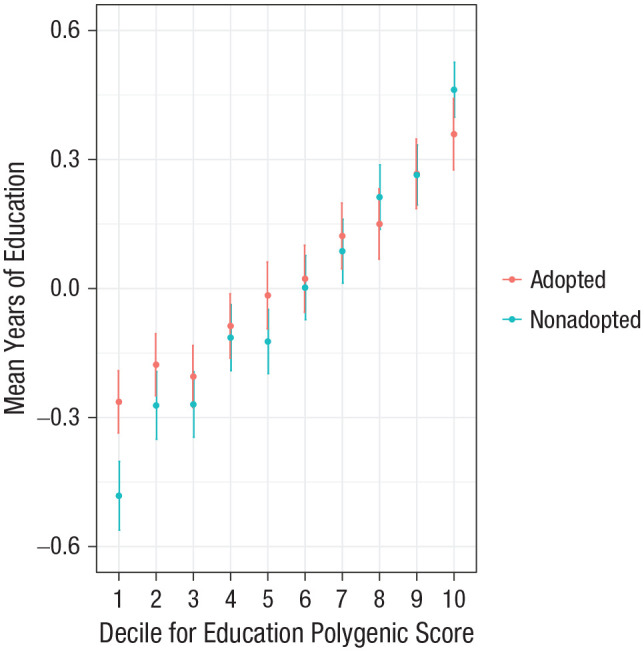
Mean years of education (standardized) per decile of polygenic score for years of education (standardized), separately for adopted and nonadopted individuals. Error bars show 95% confidence intervals.

To further explore the interaction, we plotted the mean years of education per decile of polygenic score for years of education, separately for adopted and nonadopted individuals. Figure 3 shows that for individuals in the lowest decile of polygenic score for education, those who were adopted as children achieved substantially higher mean years of education (−0.24, *SE* = 0.03; standardized) compared with nonadopted individuals (−0.40, *SE* = 0.03). This mean difference between adopted and nonadopted individuals was significant in the bottom decile (*p* = 7.05 × 10^−5^) but not for other deciles of polygenic load. See Table S4 in the Supplemental Material for full results of the decile analysis.

#### Qualitative differences in genetic influences according to adoptee status

We found that largely the same genetic influences were operating on education regardless of adoption status. First, the genetic correlation between years of education in adopted and nonadopted individuals was not significantly different from 1, 0.81 (*SE* = 0.21). Second, we found no evidence that educational attainment was associated with different traits in individuals who were adopted. Figure S1 in the Supplemental Material presents estimates of genetic correlations between years of education and 247 external traits, separately for the adopted and nonadopted samples. None of these were significantly different between adoptees and nonadopted individuals after multiple-testing correction. Because of the relatively small sample of adopted individuals, these results should be interpreted with caution.

#### Year-of-birth stratification analysis

Our final sensitivity analysis tested for differences according to year of birth in polygenic prediction from direct effects (indicated by the variance explained in the adoptees) versus from passive gene–environment correlation (indicated by the difference in variance explained between nonadopted individuals and adoptees). We found small, nonsignificant differences in the variance explained by polygenic scores for education depending on the year-of-birth group considered. Figure S2 in the Supplemental Material shows that polygenic prediction remained generally stable for the adoptees across generations at approximately 0.04, and any differences between age strata were nonsignificant. We note that subsampling reduced the statistical power to detect differences within and between groups across time. See Table S6 in the Supplemental Material for sample sizes of each year-of-birth group.

## Discussion

Cumulatively, our findings suggest that the family environment provided by relatives plays an important role in the manifestation of genetic effects on education. Polygenic scores explained significantly less variance in the educational attainment of individuals who were adopted away from their parents as children (*R*^2^ = .04) compared with individuals reared with their relatives (R^2^ = .07; difference test: *p* = 8.23 × 10^−24^). The variance explained by polygenic scores in years of education in adoptees (R^2^ = .04) approximated the prediction from the direct effects of individuals’ own DNA. The difference between the variance explained in nonadopted individuals and adoptees suggests that about half of the predictive power of polygenic scores for educational attainment comes from passive gene–environment correlation. We also found that individuals in the lowest decile of polygenic score attained significantly more years of education if they were adopted.

By showing that polygenic scores for education are twice as powerful in nonadopted individuals compared with adoptees, we suggest that genetic influence on educational attainment is magnified when individuals are reared by their close genetic relatives, with whom they share both genes and environments. Our results agree with recent evidence showing that the effects of passive gene–environment correlation reduced the variance explained by polygenic scores by 30% to 50% (Kong et al., 2018; Selzam et al., 2019).

Following other recent research (Kong et al., 2018; Selzam et al., 2019), we use the term *direct genetic effect* to refer to the effect of the polygenic score among adoptees after analyses control for passive gene–environment correlation. However, it cannot be assumed that estimating the direct effect of a polygenic score is tantamount to isolating a straightforward purely genetic effect or a genetic propensity. Genetic effects are never truly direct but are always behaviorally mediated and expressed in the context of an environment. Active and evocative gene–environment correlation mechanisms are essential in how genes influence traits in everyone, including adoptees (Plomin, 2014), and these are included in estimates of direct genetic influence.

Our observation that individuals in the lowest decile of polygenic score for education attain significantly more education if they are adopted could be due to educationally supportive adoptive environments. This agrees with previous evidence showing that adoptees had higher school achievement and intelligence-test scores than nonadopted siblings or peers who stayed with their birth family (van IJzendoorn, Juffer, & Poelhuis, 2005) and that such advantages are retained in their adult qualifications (Maughan, Collishaw, & Pickles, 1998). The specificity of this result to adoptees in the lowest decile of polygenic score links to previous evidence that the “boosting” effect may be stronger in higher-socioeconomic-status adoptive families and for children rescued from poverty (Duyme, Dumaret, & Tomkiewicz, 1999; Turkheimer, 1991). This environmental effect of adoptive parents might suggest that efforts to help individuals stay in education can be effective for those with less genetic propensity for education.

These results should be viewed in light of several limitations. First, interpreting genetic influence in adoptees as direct and free of passive gene–environment correlation requires that close biological relatives were not involved in the education of the adoptees. Unfortunately, the UK Biobank contains no information about the age of adoption beyond that it occurred in childhood, nor does it contain information about whether individuals were adopted by relatives or were able to identify and contact their biological parents. This knowledge would have allowed us to exclude individuals who were not solely socialized with adoptive families and therefore to make a precise comparison with individuals who were reared with their birth parents. However, polygenic prediction of education still differed markedly between the two groups, even though adoptees may have been in contact with their biological relatives. Thus, the effects of passive gene–environment correlation may contribute even more than half of the predictive power of polygenic scores for education, as we estimated here.

A second caveat is lack of generalizability. The UK Biobank is not representative of the general population, because there is healthy and wealthy volunteer selection (Fry et al., 2017; Keyes & Westreich, 2019), and we have analyzed data only on individuals with European ancestry. Adoptees may not be random samples of the population. Indeed, adoption status is not a purely random environmental exposure but is slightly heritable (our estimate is 6%), and this may confound our between-groups comparisons. Moreover, because detailed adoption data are lacking in the UK Biobank, we were unable to conduct sensitivity analyses adjusting for aspects of adoption that have known associations with important outcomes, including school performance. For example, it would have been useful to know whether adoptions were domestic or international or occurred in the context of childhood adversity or institutionalization (Howard, Smith, & Ryan, 2004).

Similarly, adoptive parents tend to differ systematically from other parents: They are likely to be more educated, more socially advantaged, and to have lower rates of psychopathology (Rutter, 2006). A recent U.S. study found that children are adopted into households that differ in average parental education compared with biological children (Domingue & Fletcher, 2019). This probably applies to the UK Biobank, although we cannot be certain because of the lack of parental data. If adoptive families are more homogeneous with respect to these characteristics, environmental variance may contribute less to differences in educational attainment among adoptees, and trait heritability estimates are consequently likely to be higher. However, the fact that lower environmental variance may act to inflate genetic influence in adoptees compared with nonadopted individuals makes our finding of significantly higher polygenic prediction in nonadopted individuals more striking. Again, the effects of passive gene–environment correlation for education may be even greater than we estimate.

There are several advantages of using the present adoption design for distinguishing direct genetic influence from passive gene–environment correlation. Unlike other methods, our approach does not require intergenerational data, which is valuable but has its own issues, such as cohort differences in genetic effects. Analyzing the adoptees in the UK Biobank also bypasses several limitations of traditional adoption studies, including low sample size and reliance on weak indirect proxies for inherited load for specific traits (birth-parent trait status rather than individual-level polygenic scores). However, future progress in understanding the mechanisms driving the transmission of educational attainment will require intergenerational, longitudinal, and genetically informative data sets, including detailed characterization of the home environment. A developmental approach is useful, because gene–environment correlations likely arise early in childhood, and there will be complex reciprocal effects across time. Researchers have already started to pinpoint genetically influenced aspects of families that are associated with children’s polygenic scores for education (Krapohl et al., 2017; Wertz et al., 2019; Wertz et al., 2018).

The evidence presented in this article highlights the importance of the family environment to causal mechanisms influencing individual differences in educational attainment. This can be through possessing genes that shape the educational environment provided for offspring that also directly influence attainment in the child or through providing an educationally supportive environment for an adopted child.

## Supplemental Material

Cheesman_Supplemental_Material_rev – Supplemental material for Comparison of Adopted and Nonadopted Individuals Reveals Gene–Environment Interplay for Education in the UK Biobank Supplemental material, Cheesman_Supplemental_Material_rev for Comparison of Adopted and Nonadopted Individuals Reveals Gene–Environment Interplay for Education in the UK Biobank by Rosa Cheesman, Avina Hunjan, Jonathan R. I. Coleman, Yasmin Ahmadzadeh, Robert Plomin, Tom A. McAdams, Thalia C. Eley and Gerome Breen in Psychological Science

## Transparency

*Action Editor:* Steven W. Gangestad

*Editor:* D. Stephen Lindsay

*Author Contributions*

T. C. Eley and G. Breen are joint senior authors of this article. R. Cheesman and G. Breen conceived and designed the study. Genotype-data quality control was conducted by A. Hunjan and J. R. I. Coleman. R. Cheesman analyzed the data and wrote the manuscript. All of the authors provided critical revisions and approved the final version of the manuscript for submission.

**Open Practices:** All data used in the present study are available to qualified researchers from the UK Biobank at http://www.ukbiobank.ac.uk/. The design and analysis plans for this study were not preregistered.
